# The influence of maternal psychosocial circumstances and physical environment on the risk of severe wasting in rural Gambian infants: a mixed methods approach

**DOI:** 10.1186/s12889-017-4984-2

**Published:** 2018-01-06

**Authors:** Helen M. Nabwera, Sophie E. Moore, Martha K. Mwangome, Sassy C. Molyneux, Momodou K. Darboe, Nyima Camara-Trawally, Bakary Sonko, Alhagie Darboe, Seedy Singhateh, Anthony J. Fulford, Andrew M. Prentice

**Affiliations:** 1 Medical Research Council Unit, The Gambia, P. O. Box 273, Banjul, The Gambia; 2 Department of Population Health, London School of Hygiene and Tropical Medicine, Keppel street, London, WC1E 7HT UK; 3 Division of Women’s Health, King’s College London, 10th floor North Wing, St Thomas’ Hospital, Westminster Bridge Road, London, SE1 7EH UK; 4 Kenya Medical Research Institute-Wellcome Trust Research Programme, P.O.Box 230-80108, Kilifi, Kenya; 5 University of Oxford, Nuffield Department of Medicine, Henry Wellcome Building for Molecular Physiology, Old Road Campus, Headington, Oxford, OX3 7BN UK

**Keywords:** Infant feeding, Severe wasting, Maternal stressors

## Abstract

**Background:**

Severe wasting affects 16 million under 5’s and carries an immediate risk of death. Prevalence remains unacceptably high in sub-Saharan Africa and early infancy is a high-risk period. We aimed to explore risk factors for severe wasting in rural Gambian infants.

**Methods:**

We undertook a case-control study from November 2014 to June 2015, in rural Gambia. Cases had WHO standard weight-for-length z-scores (WLZ) < −3 on at least 1 occasion in infancy. Controls with a WLZ > −3 in the same interval, matched on age, gender, village size and distance from the clinic were selected. Standard questionnaires were used to assess maternal socioeconomic status, water sanitation and hygiene and maternal mental health. Conditional logistic regression using a multivariable model was used to determine the risk factors for severe wasting. Qualitative in depth interviews were conducted with mothers and fathers who were purposively sampled. A thematic framework was used to analyse the in-depth interviews.

**Results:**

Two hundred and eighty (77 cases and 203 controls) children were recruited. In-depth interviews were conducted with 16 mothers, 3 fathers and 4 research staff members. The mean age of introduction of complementary feeds was similar between cases and controls (5.2 [SD 1.2] vs 5.1 [SD 1.3] months). Increased odds of severe wasting were associated with increased frequency of complementary feeds (range 1–8) [adjusted OR 2.06 (95%: 1.17–3.62), *p* = 0.01]. Maternal adherence to the recommended infant care practices was influenced by her social support networks, most importantly her husband, by infant feeding difficulties and maternal psychosocial stressors that include death of a child or spouse, recurrent ill health of child and lack of autonomy in child spacing.

**Conclusion:**

In rural Gambia, inappropriate infant feeding practices were associated with severe wasting in infants. Additionally, adverse psychosocial circumstances and infant feeding difficulties constrain mothers from practising the recommended child care practices. Interventions that promote maternal resilience through gender empowerment, prioritising maternal psychosocial support and encouraging the involvement of fathers in infant and child care promotion strategies, would help prevent severe wasting in these infants.

**Electronic supplementary material:**

The online version of this article (10.1186/s12889-017-4984-2) contains supplementary material, which is available to authorized users.

## Background

Severe wasting affects 16 million under 5’s worldwide and carries an immediate risk of death [1, 2]. Survivors suffer significant short- term and long- term health issues, and psychosocial and economic consequences that are often intergenerational [3–6]. The prevalence of wasting remains unacceptably high in sub Saharan Africa, with rates approaching 1–2% in the West African region [7]. In low income settings, growth faltering starts in early infancy and children can accumulate up to 80% of their total growth deficit in weight at 3 years in the first 12 months of life [8–10]. Attributable factors include poor maternal health, nutrition and socio-economic status, and infant factors including inadequate dietary intake and recurrent infections that could indicate sub-optimal child care practices [1, 11–13]. It has been estimated that, with optimal coverage, a combination of nutrition specific and nutrition sensitive interventions could reduce under 5 deaths by 15% in low income settings [14–16]. Unfortunately, the delivery platforms for these evidence based interventions are often under resourced, and when combined with low uptake, result in poor coverage with limited impact on maternal and child undernutrition [15, 17]. Scaling up of nutrition sensitive interventions has the potential to enhance progress in childhood undernutrition [17].

There is a growing recognition that maternal common mental health disorders (CMDs) including depression and anxiety impair parenting skills, shorten duration of breastfeeding and are important determinants of growth and health outcomes of children in low income countries [18–20]. However, there have been conflicting findings on the association between maternal depression and child undernutrition in LMICs, with an overall stronger association in South Asia compared to Africa [19–21]. A meta-analysis of studies done in 11 LMICs showed that children of mothers with depressive symptoms were more likely to be underweight or stunted [22]. Sub analysis of three longitudinal studies showed a stronger effect for both underweight and stunting [22]. Notably, wasting was not used as an outcome measure for undernutrition in these studies. However, more recently, Ashaba et al. found that in their inpatient nutrition rehabilitation unit in southwest Uganda, maternal depression was significantly associated with severe wasting [13].

The Gambia, where half of the population is rural, was one of the few African countries to meet the fourth Millennium Development Goal of reducing under 5 mortality by two thirds from 1990 to 2015 [23]. Unfortunately, over this interval the prevalence of wasting in this age group remained unchanged at 10% [23, 24]. Previous studies from rural Gambia found that undernutrition was associated with inappropriate infant feeding practices and the acquisition of enteric infections in early childhood, whilst living with maternal grandmother was protective [25–28]. In addition, poverty, cultural beliefs about malnutrition, lack of support networks and gender inequality were found to be determinants of inappropriate child health and nutrition practices [29]. To our knowledge no previous studies have explored the association of maternal psychological status and severe wasting in infants, in the context of adverse social circumstances such as poverty, food insecurity and gender inequality in The Gambia. This study therefore aimed to explore the maternal and infant health, psychosocial and environmental factors that are associated with severe wasting in rural Gambian infants, in order to identify targets for intervention that would contribute to scalable nutrition interventions.

## Methods

### Study design

The study utilised a mixed methods approach involving both quantitative and qualitative methods. In the quantitative methods, we used a case-control study design among children who had been enrolled into the Early Nutrition and Immune Development (ENID, Trial registration number: ISRCTN49285450, Retrospectively registered Sept. 12, 2009) randomised trial (details in Additional file 1) [30]. The quantitative phase aimed to identify and quantify the risk factors associated with severe wasting in infancy in this population. In the qualitative phase, a descriptive-exploratory approach was used to explore constraints, knowledge, attitude and practice of infant rearing and feeding among carers. The use of a mixed method approach evolved out of the need for an interdisciplinary approach to address complex health problems such as severe wasting in infancy, that quantitative or qualitative approaches alone were not able to adequately address [31].

### Setting

This study was undertaken in the West Kiang district of rural Gambia that contains 36 villages of varying sizes with a total stable population of almost 15,000, of whom 2300 (15%) are children under 5 years of age [32]. The main income generating activity is subsistence farming, but over the past decade this district has been prone to food insecurity due to erratic rainfall patterns, necessitating emergency relief food supplies [33, 34]. The climate has a long dry ‘harvest’ season (*November–May) and a short wet ‘hungry’ season (late June to mid-October), when agricultural work, depletion of food supply prior to harvest and infectious diseases peak* [35]*.* Breastfeeding from birth to 2 years is the norm and complementary feeds are often nutritionally inadequate [36].

Mandinka are the predominant ethnic group, but there are also other ethnic groups including Fula [32]. The majority of the population are Muslims but traditional African beliefs are an important part of their spiritual lives. The bulk of domestic duties, agricultural work and rearing of children is undertaken by the women, who are also often subjected to domestic violence [23]. An increasing number of children including girls are accessing primary school education, but in the local government area that includes the West Kiang district, only 42% transition to secondary school [23]. The literacy levels, particularly amongst women in rural areas remain low and a large proportion of the population live below the moderate poverty line of less than US$ 2/ day [23, 33].

The UK Medical Research Council (MRC) has been providing free comprehensive primary health care services including antenatal and child health clinics, for over 40 years to 3 rural villages in the West Kiang district: Keneba, Manduar and Kantong Kunda [32, 35]. This has contributed to greater than 80% decline in both the infant and under 5 mortality rates in these 3 villages over the past 4 decades [35], well ahead of the Gambian national estimates [23]. Unfortunately, the patterns of undernutrition in children in this population have not followed a similar trend, and at 2 years of age 11% are wasted, 22% are underweight and 30% are stunted [24], in line with the national estimates [23]. In recent years, the West Kiang Demographic Surveillance System (DSS) has been established that includes all the 36 villages (including Keneba, Manduar and Kantong Kunda). The inhabitants in all these villages can access free health care services at the MRC Keneba clinic (outpatient antenatal, postnatal, child health and adult services), but continue to have their routine antenatal and child health checks at their local health care facilities run by the Ministry of Health in The Gambia [32].

### Sampling and study population

#### Quantitative

##### Sample size

Our sample size calculations were based on analysis of the post-matching variables (in this case those derived from the questionnaire that include access to clean drinking water, hand washing habits and sanitation; infant feeding practices; socio-economic status, family structure and maternal depression) using the normal approximation to the binomial and assuming a case-control design (Additional file 1: Table S1). Our power calculations showed that 97 cases and 291 controls would be sufficient to identify, with 90% certainty at the 5% level of significance, factors for which the odds ratio of becoming severely malnourished is 2.13 or more.

##### Inclusion and exclusion criteria

All the children who had ever been in the ENID trial were eligible. The weight-for-length z-scores (WLZ) for the infants were calculated using WHO Anthro (version 3.2.2, January 2011) and macros according to the WHO 2006 growth reference standards [37]. Cases were all the children who had a WLZ < −3 i.e. were severely wasted, on at least 1 occasion between 0 and 12 months of age (excluding the first week of life that would have been neonates with intrauterine growth restriction). Controls were all the appropriately matched children with a WLZ > −3 at the age when the cases had a WLZ < −3. The controls were matched based on age (i.e. similar date of birth (+/− 1 month), gender, village size (large [>750 inhabitants], medium [250–750 inhabitants] and small [<250 inhabitants]) and distance from the MRC clinic in Keneba (binary near [<19 km]-far [≥19 km]). The ENID trial excluded all multiple pregnancies, infants exposed to HIV and those with congenital anomalies [30, 38].

Eighty-nine children were identified as having a WLZ < −3, but we excluded one as their WLZ was clearly an error. Only 77 could be appropriately matched to controls. Sixty-one were matched 1:3, five were matched 1:2 and 11 were matched 1:1 giving a total of 280 children (78% of the expected sample size). Seventy-two (94%) of the cases and 200 (99%) of the controls were alive at the time of this follow-up study (Table 1).

**Table 1:** Comparison of characteristics between cases and controls

Characteristics	Cases *N* = 77	Controls *N* = 203	*P* value
Demographic and anthropometric characteristics			
*Children*			
Parity, median (IQR)	4 (3, 7)	4 (2, 6)	
WLZ at growth faltering time point in cases, mean (SD)	−3.6 (0.5)	−0.6 (1.1)	**<0.005** ^**a**^
WLZ at 12 months, mean (SD)¥	−2.1 (1.3)^b^	−0.8 (1.1)^c^	**<0.005** ^**a**^
Age in years at time of interview, mean (SD)	2.9 (0.8)	2.9 (0.8)	
Male, *n* (%)	48 (58)	134 (61)	
One or more children under 5y in household (other than index child) *n* (%)	75 (97.4)	196 (96.6)	
*Carers*			
Paternal age, mean, SD	47.3 (9.7)	48.7 (12.1)	
Maternal age, mean, SD	35 (6.4)	34 (6.7)	
Maternal depressive symptoms, n (%)	10 (13)	36 (12.4)	
Carer from 0 to 12 months- mother, n (%)	77 (100)	199 (98)	
Mothers have freedom to move around without escort	76 (98.7)	198 (99.5)	
*Environmental*			
Distance from MRC clinic in km, median (IQR)	17 (10, 23)	13 (5, 23)	
Population size of village, median (IQR)	768 (528, 1265)	768 (610, 1265)	
Socioeconomic status			
*Education level of mother*			
No formal education	23(30)	40 (20)	
Arabic school	40 (52)	122 (61)	
Less than primary school	8 (10)	24 (12)	
Completed primary school	6 (8)	9 (5)	
Completed secondary school	0	4 (2)	
*Mother’s income*			
Farming	65 (86)	163 (82)	
Business	4 (5)	7 (3)	
Salary	2 (3)	1 (1)	
Other	5 (6)	28 (14)	
Guaranteed monthly income	6 (8)	10 (5)	
*Housing status*			
Number of rooms in for sleeping household, median (IQR)	2 (2,3)	2 (2,3)	
Number of people in household past 6 months, median (IQR)	6 (4,8)	5 (4,7)	
*Water and sanitation*			
Toilets, n (%)¥			
Flushing	0 (0)	1 (<1)	
VIP latrine	1 (1)	2 (1)	
Traditional pit latrine	70 (92)	190 (95)	
No toilet	5 (7)	5 (3)	
Water source past 6 m, *n* (%)			
Piped water in house	0 (0)	1 (<1)	
Covered public well	0 (0)	3 (2)	
Public tap	69 (91)	173 (87)	
Open public well	5 (7)	10 (5)	
Open well in compound	0 (0)	1 (<1)	
Deep tube well	2(3)	11 (6)	
*Accessibility to water, n (%)*			
Less than 30 min	75 (97)	192 (96)	
30 min or more	2 (3)	7 (4)	
Ability to fetch drinking water daily, *n* (%)	75 (97)	196 (98)	
Number of daily trips for water, median (IQR)	5 (4,6)	5 (4,6)	–
Water purification for drinking water done	26 (34)	76 (38)	
*Water purification method, n (%)¥*			
Filtration through cloth	25 (96)	76 (100)	
Filtration through ceramic	1 (4)	0 (0)	
*Principal component analysis 1*			
Wealth quintiles, *n* (%)			
1 (Poorest)	30(39)	63 (31)	
2	19 (25)	54 (27)	
3	1(<1)	8 (4)	
4	11 (14)	41 (20)	
5 (Wealthiest)	16 (21)	37 (18)	
Infant feeding and care practices			
*Breastfeeding and complementary feeding*			
Ever breastfed¥	76 (100)	194 (100)	
Age breastfeeding stopped, months, median (IQR)	24 (20, 24)	24 (20, 24)	
Age of introducing complementary foods, months, mean (SD) (prospective data collection)	5.2 (1.2)	5.1 (1.3)	
Commonest complementary food-coos (maize meal) porridge, *n* (%)	54 (65.1)	155 (70.1)	
Mode of feeding-spoon *n* (%)	75 (97)	198 (98)	
Frequency of complementary feeds, mean (SD)	4 (1.0)	3 (0.9)	
*Frequency of feeding per day, n (%)*			
X1	0 (0)	1 (1)	
X2	6 (7)	17 (8)	
X3	26 (34)	114 (58)	
X4	30 (40)	46 (23)	
X5	11 (15)	15 (8)	
Greater than X5	3 (4)	5 (2)	
Scheduled feeding, *n* (%)	52 (70)	142 (72)	
Own bowl at feeding time, *n* (%)	75 (97)	198 (98)	
*Decision-maker for feeding of infant*			
Mother alone	68 (90)	188 (95)	
Mother and father	6 (7)	8(4)	
Mother and mother in law	2 (3)	1 (<1)	
Mother and other	0 (0)	1 (<1)	
*Decision-maker for medical care of infant*			
Mother alone	51 (67)	135 (68)	
Mother and father	23 (30)	60 (30)	
Mother and mother in law	2 (3)	3 (2)	
Mother and other	0	1 (<1)	
*Hand washing ¥*			
Water alone	7 (9)	17 (9)	
Water and soap	70 (91)	181 (91)	
Water and mud or clay	0 (0)	1 (<1)	
Health status of infants and siblings			
*Illness episodes in index child*			
Diarrhoea, median (IQR)	2 (1, 4)	2 (1,4)	
Morbidity, median (IQR)	10 (6, 14)	9 (6,13)	
Number who died in first 12 months, *n* (%)	4 (5)	2 (<1)	
Number died after 12 months, n (%)	1(<1))	1 (<1)	
*Sibling*			
No sibling deaths, *n* (%)	22 (29)	65 (32)	
Age in months of sibling who most recently died, median (IQR)	6 (2, 7)	2 (1, 5)	

^a^ Two sample Student T-test ¥Missing data

^b^*N* = 53

^c^*N* = 169

#### Qualitative

**Fig. 1:**
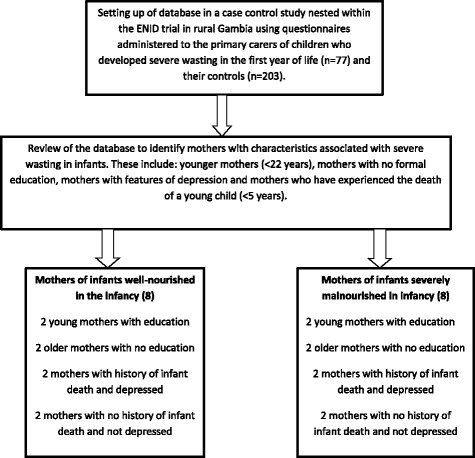
Sampling framework for qualitative data

Based on the preliminary analysis of the quantitative data, maternal illiteracy, history of a child death and maternal depression were associated with severe wasting in infancy. We therefore used these findings to develop a sampling framework for the in-depth interviews (IDIs) (Fig. 1). We used stratified purposive sampling where mothers of infants who were in both groups i.e. cases and controls, were selected, in order to capture a diversity of views and experiences about psychosocial, cultural, economic and environmental factors that influenced their infant feeding and rearing practices and their perceptions on how these impacted on the nutritional status of their children. It also allowed us to look for any major differences across the 2 sets of carers i.e. those with or without severely malnourished children.

Purposive sampling is commonly used in qualitative research to identify a particular group of people who possess certain characteristics or who reside in circumstances pertinent to the phenomenon being studied and are therefore “information-rich” [39]. Stratifying the sampling framework also allowed us to identify any major variations in practices or perceptions within each strata [39]. This iterative process led us to undertake IDIs with fathers of some of the cases and controls, and with the research staff. We stopped recruiting participants for IDIs when we got to the point of “data saturation” i.e. the point in data collection and analysis when “new information does not generate any new themes or variability within the themes in the dataset” [40]. In total, we undertook 19 IDIs with carers (8 with mothers and 2 with fathers of cases, 8 with mothers and 1 with a father of controls) and 4 with the research staff (3 male, 1 female).

##### Data collection

**Fig. 2:**
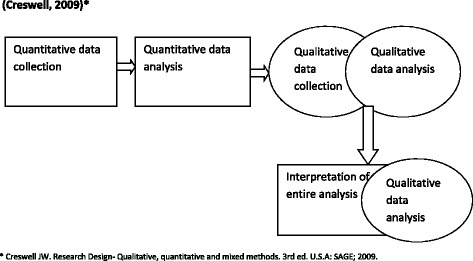
Sequential explanatory strategy for data collection model

We used the sequential explanatory strategy for data collection as illustrated in Fig. 2 [31]. This involved quantitative data collection and preliminary analysis to guide the development of the qualitative sampling framework, in the first phase followed by qualitative data collection and analysis in the second phase [31].

#### Quantitative

Baseline maternal demographic data including maternal age, parity, residence, marital status, socio-economic status (assessed using household income, maternal education, household assets, housing quality and access to water and sanitation) and details of her spouse were collected at recruitment into the ENID trial [38]. In addition, data on the infant anthropometry, feeding and illness episodes were collected prospectively during the trial [30].

##### Questionnaires

From November 2014 and March 2015, we administered two questionnaires to the main carers of the infants. The first one addressed questions about the demographics and socioeconomic status; infant feeding (type and duration of breastfeeding and complementary feeding practices including the timing of introduction); and water, sanitation and hygiene (WASH) practices (access to sanitation and drinking water). The second one was a modified version of the Edinburgh Depression Scale (EDS) that was used to assess symptoms of depression in the mothers. This has 10 items and is a validated screening tool for depression in postnatal and non-postnatal women in a wide range of settings worldwide, including West Africa [41–43]. This tool was translated into Mandinka according to the principles of the WHO translation protocol [44]. Further details about the tools can be found in the Additional files 2 and 3. One trained male senior field worker administered the modified EDS closely supervised by HMN. Women who had a modified EDS ≥ 12 (total score 30) were classified as being depressed, and were referred to the primary health care services at MRC Keneba clinic for counselling by trained nurse counsellors and/or assessment by the doctors in the clinic, who were experienced in managing depression.

##### Anthropometry and infection episodes

The collection of anthropometric data and infant infection episodes for the ENID Trial has been described in detail elsewhere [30, 36]. In summary, the measurements in the infants including weight, height, were performed by trained senior midwives and field workers. Lengths were measured on a Raven Kiddimetre® (Raven Equipment, Great Dunmow, Essex, UK) to the nearest 0.1 cm. Weight was measured on minimally clothed infants and recorded to the nearest 10 g using electronic Seca 336 scales that were calibrated regularly. Infants had their measurements done at birth (within 72 h of delivery) and study visits on weeks 1, 8, 12, 16, 24, 40 & 52. All infants had a weekly visit from the trial field workers who recorded all their illness episodes including diarrhoea and chest infections during that interval.

#### Qualitative

The development of the qualitative data collection tools (i.e. observation tool and IDI guide) was an iterative process that involved the revision of the tools in order to facilitate further exploration of factors that were found to be significantly associated with severe wasting in the infants in this study (i.e. increased frequency of complementary feeds) in the quantitative data analysis. We also added probing questions to explore the reasons for why certain factors that we had hypothesised as being important contributors to severe wasting in this population were not found to be so in the quantitative analysis.

##### Informal observations

During informal observations, researchers systematically watch people and events to observe everyday behaviours and relationships [45]. This enables the researcher to understand the perspectives of the study population and their physical, socio-cultural and economic context [45]. Our female study nurse undertook these informal observations systematically using an observation tool, 1 week prior to the respective IDIs. During these visits, she systematically observed and documented details of the home environment. These included the daily activities of the mothers, with a focus on their child rearing practices including the preparation of food for the infants or children in the household, hygiene and sanitation practices. She also observed the dynamics within families – non-verbal communication and the levels of interaction between the mothers and other members of the family e.g. the father, the index child, the grandparents and co-wives. These observations provided a rich source of information about the context prior to the IDIs whilst enabling the study nurse to develop a rapport with the mothers that enhanced the conduct of the interviews.

##### In-depth interviews

In these sessions one interviewer interviews only one person [39]. The IDIs were done between April and July 2015. Our female study nurse conducted the IDIs with carers in Mandinka and Fula. They were all undertaken in the respondents’ compounds, which was their preference. HMN conducted the IDIs with the research staff in English. The IDI guide was divided into the following four areas of inquiry: infant feeding; parenting skills; hygiene; and mental health/learning difficulties. Each section consisted of a combination of structured and open ended questions. We asked all respondents the same questions in a similar sequence, and the interviewer probed inductively on relevant responses. The interviews were audio recorded and the interviewers took field notes for all the interviews.

The interviews in Mandinka were transcribed by a trained independent transcriber, and those in Fula by a field worker from the study team, who had previous experience of transcribing qualitative interviews. This concurrent transcription and translation of these interviews was largely due to the limited time and resources available to undertake a two-stage process of transcribing in the local languages, then translating. In addition, they are not formally written languages which would add to the difficulty. The study nurse reviewed the transcripts for translation accuracy and discussed these with HMN during debriefing sessions. Where the translation was problematic, we compared the transcripts to the original audio recordings and revised them accordingly. In addition, two randomly selected transcripts were translated and transcribed by a field worker, who was a member of the research team and we found that there was agreement with the original transcriptions. This helped to establish both the validity and reliability of the transcripts [46]. HMN also conducted IDIs with the research team in order to explore their perceptions on the risk factors for SAM, as Gambian health care workers and field staff with over 2 years of experience of working in this community were. In addition, we used it to validate the findings from the IDIs with the carers.

### Data analysis

#### Quantitative

Data were analysed using Stata (version 12.0, StataCorp, College Station, TX). We incorporated available prospectively collected data for the following variables: infant feeding data on timing of the introduction of complementary feeds, parity, infant morbidity and wealth indices. Descriptive statistics were computed to report number and percentage for categorical data, and the mean or median and standard deviation (SD) or interquartile range (IQR) for continuous data. The two sample Student T-test was used to assess the difference WHZ at the point when the cases growth faltered and at 12 months of age between the cases and controls. A principal component analysis (PCA) was used to determine household socioeconomic status using an asset based index in which we included the following six socioeconomic indicators: ownership of television, car, electricity, motorcycle, bicycle, or animal cart (yes/no), that were prospectively collected in the ENID trial. The adequacy of these indicators was assessed for inclusion in the PCA using the Kaiser-Meyer-Olkin measure [47], and they explained over 22% of the variability in the combined socioeconomic factor score (Further details in Additional file 1). The households that the children came from were classified into quintiles based on Filmer and Pritchett’s method [48].

The association between the demographic, psychosocial, infant feeding and WASH characteristics and severe wasting in infancy was assessed using conditional logistic regression, where cases were only compared to controls in the same set for crude odds ratios (ORs) and then, adjusted for confounders (including maternal age that was correlated to both severe wasting and maternal education/parity/number of child deaths; and paternal age that was correlated to both severe wasting and socioeconomic status) and collinearity in the multivariable model [49]. Using a backward stepwise model selection criteria [49], where explanatory variables with a sparse amounts of data (e.g. sibling deaths) and those with unreliable estimates where the 95% confidence intervals were ≥10, were dropped from the model. In addition, we incorporated the prospectively collected data variables where the retrospectively collected data was either not available or was not robust e.g. age of introduction of complementary feeds and household assets in order to improve the accuracy of our findings.

The final conditional regression model had 18 explanatory variables. This enabled us to estimate the effect of these exposures on severe wasting in infancy through computing adjusted ORs. We used 95% confidence intervals (CI) and *p*-value <0.05 to determine statistical significance. Our purpose was exploratory rather than intended as a definitive test of a particular hypothesis.

#### Qualitative

In qualitative research data analysis is an inductive process that seeks to learn about the perceptions that participants hold about a problem or issue by identifying patterns or themes [50]. HMN used the information from the informal observations, interview transcripts and summary information from the debriefing sessions, to develop summary sheets of caregivers in both groups. She then used headings and sub-headings based on the study questions and emerging themes to categorize information from the summary sheets. These ensured that we could comprehensively describe each group of mothers. This process formed the preliminary stages of data analysis as it involved reading and re-reading of the observation tools, interview transcripts and field notes, and thorough familiarization with the data from each caregiver.

**Fig. 3:**
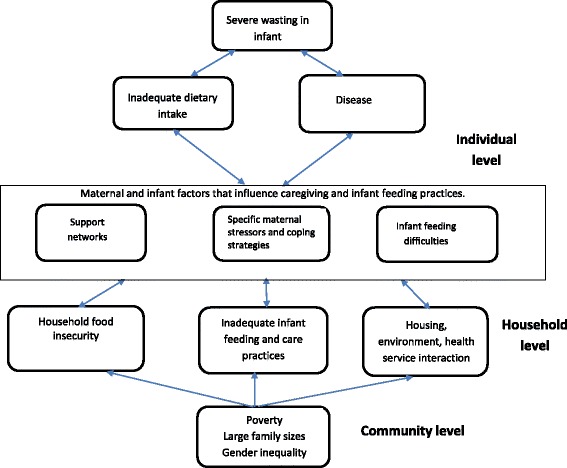
Conceptual framework of maternal factors that influence infant nutritional status

We undertook data analysis alongside data collection in order to allow questions to be refined and new avenues of inquiry to develop [51]. We used NVivo 10 software (QSR International Pty Ltd. 2012) in subsequent data management and analysis using codes. After extensive familiarization with the data, HMN developed a coding scheme [52]. Two of the authors (HMN, MKM) agreed on the coding scheme who together reviewed the first 10% of respondents’ transcripts independently and through multiple iterations came to an eventual consensus. We revised the coding scheme further during the analysis. These multiple iterations are a key inductive process in the development of data coding schemes [53]. HMN undertook the NVivo coding of all the 23 transcripts. Using a thematic analysis approach, we grouped the data into themes and sub-themes and evaluated key emerging themes and how the themes/subthemes were interconnected [54–56]. To guide our analysis, we used a conceptual framework (Fig. 3) that was based on the UNICEF Conceptual framework for undernutrition in children (Additional file 1) [11].

## Results

### Quantitative

#### Comparison of characteristics of cases and controls

The mean WLZ for the cases and controls were −3.5 (SD 0.5) and −0.6 (SD 1.1), *p* < 0.005 respectively at the age they were matched. The mean WLZ of the cases and controls at 12 months remained significantly different (−2.1 [SD 1.3] and −0.8 [SD 1.1], *p* < 0.005). There two groups were similar with regard to age, gender, distance from the MRC clinic and village size. At the start of this follow up study, all the children were over 6 months of age with a mean age of 2.9 years (SD 0.8) for cases and controls. The primary carers during the first 12 months for all the children in the study were their mothers (Table 1).

#### Maternal and socioeconomic factors

The overall prevalence of maternal depressive symptoms was 13% and this was similar between the mothers of the cases and of the controls (13% vs 12%). There maternal level of education was also similar between cases and controls and the majority had attended Arabic school (Islamic studies) (Table 1).

Overall traditional pit latrines were the commonest form of sanitation within compounds (95% of participants) and the source for clean drinking water was less than 30 min away for most mothers (>96%). Only a third of mothers undertook water purification and this was often filtration using a cloth and over 90% reported that they practised hand washing with soap during their daily activities (Table 1).

#### Infant feeding

Infant feeding choices were often made by the mothers. Infants were often breastfed up to 24 months, with the introduction of complementary feeds at an average of 5.2 months. The commonest complementary feed was maize meal (“coos”) porridge. Infants were often fed 3 complementary meals a day and mothers in both groups most frequently reported practising scheduled complementary feeding (70% cases vs 72% controls). The mothers of the cases reported a higher frequency of complementary feeding (mean 4 vs 3) (Range 1–8) (Table 1).

#### Illness episodes in infancy

Overall children in both groups had a median of 2 (IQR 1, 4) diarrhoea episodes in the first 12 months of life and this was similar for cases and controls (10 [IQR 6, 14] and 9 [IQR 6,13] (Table 1).

#### Sibling deaths

Overall, a third of mothers had experienced the death of a child other than the index child, with the most recent loss being closer to the neonatal period in the controls (median 2 [IQR 1, 5] vs 6 [2, 7] months (Table 1) (Further details in Additional files 1, 2, 3, 4 and 5).

#### Risk factors for severe wasting

In the univariable analysis, the only factor that was associated significantly with severe wasting in the infants was increasing frequency of complementary feeds (OR 1.51 [1.13–2.01], *p* = 0.005) (Table 2). In the multivariable model, this association remained significant (OR 2.06 (1.17–3.62), *p* = 0.01). The treatment of water showed a trend towards a protective effect but this was not statistically significant (OR 0.31 (0.09–1.04), *p* = 0.06). Maternal depressive symptoms were not significantly associated with severe wasting in infants in this model (OR 1.37 (0.32–6.00), *p* = 0.67) (Table 3).

**Table 2:** Univariable analysis of risk factors for severe wasting in infants

Variables	Unadjusted OR (95% CI)	*P* value
*Carer factors*		
Current carer	1.03 (0.30–3.46)	1.00
Mother	1.00	
Father lives in household	0.73 (0.44–1.22)	0.23
Father not in household	1.00	
Carer education	0.79 (0.52–1.20)	0.33
No carer education	1.00	
Maternal age	1.02 (0.98, 1.06)	0.34
Maternal age 21 years	1.00	
Paternal age	1.00 (0.96–1.01)	0.48
Paternal age 25 years	1.00	
No monthly income for mother	2 (0.65–6.12)	0.23
Monthly income for mother	1.00	
Children under 5y in household	1.39 (0.96–2.01)	0.09
No children under 5y	1.00	
Maternal depressive symptoms	1.18 (0.54–2.61)	0.68
No maternal depression	1.00	
Other makes decision to seek medical care	1.09 (0.62–1.90)	0.78
Mother makes decision to seek medical care	1.00	
Principal component 1 (socioeconomic measure)	1.07 (0.89–1.29)	0.48
Lowest quintile	1.00	
Principal component 2 (socioeconomic measure)	0.86 (0.68–1.10)	0.19
Lowest quintile	1.00	
*Infant factors*		
Parity	1.01 (0.90–1.14)	0.82
Index child only child	1.00	
Age breastfeeding stopped	1.0 (0.93–1.10)	0.93
0 months	1.00	
Age of introducing complementary feeds (prospective)	1.09 (0.86–1.39)	0.47
1 month	1.00	
Other decides on type complementary food for infant	1.09 (0.62–1.90)	0.78
Mother decides on type of complementary food for infant	1.00	
Frequency of complementary feeds (1–8)	1.51 (1.13–2.01)	**0.005**
Feeds once a day	1.00	
Other feeds infant	0.81 (0.26–2.50)	0.72
Mother feeds infant	1.00	
No scheduled feed	1.11(0.61–2.04)	0.74
Scheduled feeding	1.00	
*Illness episodes in infancy*		
Diarrhoea episodes	0.96 (0.84–1.09)	0.51
No diarrhoea episodes	1.00	
Morbidity	1.02 (0.96–1.07)	0.53
No morbidity	1.00	
*Sibling factors*		
Sibling death	1.04 (0.55–1.94)	0.91
No sibling death	1.00	
*Environmental factors*		
Number of people sleeping in household	1.90 (0.99–1.21)	0.09
2 people	1.00	
Number of rooms in household	1.24 (1.00–1.60)	0.10
1 room	1.00	
Source of water more than 30 min Source of drinking water less than 30	0.75 (0.14–4.00)	0.74
minutes	1.00	
Drinking water for household fetched daily Drinking water for household not fetched	0.59(0.10–3.56)	0.57
daily	1.00	
Increasing trips for fetching drinking water	0.94 (0.82–1.10)	0.47
One water trip	1.00	
Water treatment	0.85 (0.48–1.50)	0.57
No water treatment	1.00	
No toilet in compound	3 (0.78–11.51)	0.11
Toilet in compound	1.00	
Hand washing without soap	1.10 (0.44–2.72)	0.84
Hand wash with soap	1.00

**Table 3:** Multivariable analysis of risk factors for severe wasting (after fitting model)

Variables	OR, 95% CI	*P* value
*Carer factors*		
Maternal depressive symptoms	1.37 (0.32–6.00)	0.67
No depression	1.00	
Maternal age	1.03 (0.91–1.18)	0.64
Maternal age 21 years	1.00	
Paternal age	0.95 (0.88–1.02)	0.15
Paternal age 25 years		
Sibling death	0.47 (0.11–2.00)	0.30
No sibling death	1.00	
Principal component 1 (lower socioeconomic measure)	1.23 (0.88–1.72)	0.23
Lowest quintile	1.00	
Principal component 2 (lower socioeconomic measure)	1.19 (0.71–1.98)	0.50
Lowest quintile	1.00	
*Infant factors*		
Parity	0.99 (0.76–1.28)	0.92
Index child only child	1.00	
Age breastfeeding stopped	1.05 (0.90–1.19)	0.53
0 months	1.00	
Age of starting complementary feeds (prospective)	0.89 (0.71–1.11)	0.30
1 month	1.00	
Frequency of complementary foods (1–8)	2.06 (1.17–3.62)	**0.01**
Feeds once a day	1.00	
No scheduled feeding	2.21 (0.56–8.64)	0.26
Scheduled feeding	1.00	
Other feeds infant	0.45 (0.05–4.00)	0.47
Mother feeds infant	1.00	
Other makes decision to seek health care	1.42 (0.47–4.34)	0.54
Mother makes decision to seek medical care	1.00	
*Illness episodes in infancy*		
Diarrhoea episodes	0.82 (0.62–1.09)	0.51
No diarrhoea episodes	1.00	
Morbidity	1.02 (0.96–1.07)	0.53
No morbidity	1.00	
*Environmental factors*		
Water treatment	0.31 (0.09–1.04)	**0.06**
No water treatment	1.00	
Drinking water for household fetched daily	0.88 (0.64–1.21)	0.44
Drinking water for household not fetched daily	1.00	
Number of people sleeping in house	1.02 (0.81–1.30)	0.85
2 people	1.00	

Adjusted for maternal age and paternal age

### Qualitative

Mothers in this study were knowledgeable about the infant feeding and rearing recommendations having received intensive and sustained health promotion messages from the ENID trial study team. The mothers who we interviewed were all married and lived in their marital homes with their husbands and parents-in-law. All the mothers experienced poverty and were financially dependent on their husbands with only a minority having accessed formal ‘English’ education. From the quantitative analysis, inappropriate infant feeding practices appeared to be a key factor in severe wasting in infants. We found that the infant feeding and rearing capacity of mothers was heavily influenced by 3 key factors: i) her social support network, most importantly her husband; ii) infant feeding difficulties; and iii) maternal psychosocial stressors.

#### Support networks

We observed that within households, mothers were preoccupied with domestic chores (including food preparation) and ‘gardening’ (farming) in order to provide food for their families. In addition, they also bore the responsibility of caring for their aging parents-in-law. Apart from mothers who were still breastfeeding, they were often unable to supervise feeding or play sessions with infants or young children and relied on a family member, e.g. paternal grandmother, or an older sibling for support with this. When these support networks were not available, these sessions were unsupervised. This lack of supervision sometimes posed a risk to the infants, e.g. being too close to a fire, or sharing a feeding bowl with a domestic animal.

*“Yes, because if the mother leaves the child at home with the younger ones who cannot take care of that child, for example...I met the child eating with a dog. You see, and whilst the elder sister was there who could not even take care of that child. When I asked, she said the mother had gone to the garden...” (Field worker, 150211_003)*

Under these circumstances, mothers were therefore unable to quantify the amount of food their infants had consumed and often assumed they were full when they stopped crying or feeding.

In this challenging context for mothers, the support of their husbands appeared to be a crucial mitigating factor. Both the male and female respondents perceived the role of the father as being a provider even when he was not permanently based in the home, i.e. bringing in resources to support the mothers’ infant feeding and rearing strategies such as purchasing ingredients for enriching complementary feeds (sugar and butter for enriching “pap” (maize meal porridge)) or equipment for maintaining hygiene such as soap, as well as transport to access health care facilities for the infant and other family members.

*“Fathers actually have a role to play, because fathers should stand in the care of both the mother and the child. Both the mother and father own the child. … father’s role is to buy soap for the mother, in order to launder and if he (infant) starts to eat, you can buy food stuff which will benefit him (infant) and give to the mother to give him. But for today if you want to leave him in the care of the mother only, “baa fele a,amang semboo soto” (the mother is not financially strong)”…. (Mother, control_MAL130J, Bajana)*

In addition, mothers also thrived on a supportive marital relationship with their husbands and this seemed to enhance their ability to make decisions in line with the infant feeding and rearing strategies. Mothers were the primary decision makers on matters pertaining to infant feeding and rearing, including accessing preventative or minor illness health care services that were often accessible on foot. This is because, their husbands, apart from those who worked for the MRC or were government health care workers (HCWs), had limited knowledge of the infant feeding and rearing guidance and therefore relied heavily on the mothers to make these choices. However, when their infants were very sick and required admission to the MRC Keneba clinic, mothers sought the financial support of their husbands for transportation.

*“Yes, if they involve themselves in, it will give better care to the children. When a mother doesn’t have something and you the father have it then you put it there. …it will also add the child care. But if the mother doesn’t have it and you (husband) too did not do it, caretaking will not take place. He (infant) will be left out to get sick.” (Mother, case_MAL019P, Kuli Kunda)*

Although, the regular pastime for men in the West Kiang is to sit in the “bantaba” (a traditional meeting place for the men of the village), when not engaged in agriculture or male-led work, some husbands recognised the domestic pressures that the mothers were under, reflected on this and considered/admitted to offering practical assistance to their wives (mothers) so that the care of the infant was optimised, despite this being culturally alien. For them the aims were to support the mother but also their infants*.*

*“Yes I (husband/father) helped the mother in that because the reason why I helped her, I like myself that is why. I know that she alone cannot do all that… If she is at one place, I can also be at the other place because to leave it with her alone, she is not a slave. We are all marriage partners. … I do not leave my wives. I always help them. And in terms of hygiene, I helped them there too. Because I clear my environment, I tell them “make this place and make the other place”. If you want the children’s health, you have to take care of your place. If you do not take care of them, there will be no health.” (Father, case_MAL008F, Jali)*

Mothers also felt that the practical involvement of their husbands in the care of their infants enhanced their nutrition. Mothers mentioned support such as feeding, bathing and taking the infant to the clinic to enabled the mother to complete her tasks and therefore had time to give appropriate attention to the feeding of their infants.

*“Yes they also have a role to play. That is because a child is closer to the wife that is why caretaking starts with the wife. But the husband also has a role. … The child’s needs should also be the role of the husband. The needs such as make him clean, and should feed him. Like in the case of feeds, whatever your earnings can allow you to afford, you should give to the child because that will improve his health.” (Mother, case_MAL015R, Jali)*

However, when this support from their husbands was not forthcoming e.g. due to abandonment of mothers and their children because of marital tension or the cultural union of widows with a close male relative; the mothers relied more heavily on the support of the maternal grandmother/parents or peers. This support was less reliable as often these parties themselves relied on financial support from elsewhere. Under these circumstances, mothers found that this constrained their ability to adhere to the infant feeding recommendations, by limiting their options for enriching the popular complementary food pap. In desperation, some mothers opted to increase the breastmilk content in the diets of their over 6-month-old infants and gave less or no complementary food, which was presumably detrimental to the nutritional status of their infants.

*“…I just noticed that when I asked him to do… he did not do it. When I ask from him once, twice and thrice, and he does not do it, I never bother myself to tell him again. When I go to my people if on a particular day they too do not have it, then I leave it. I do not cook for him on that day and keep it for him, I then breastfeed him.” (Mother, case_MAL019P, Kuli Kunda)*

*“You see a man; he may sit without digging toilet at home. He will not buy soap and will not buy anything. You will be responsible of putting everything in your food. You will buy soap and there is no toilet in the compound. That can bring difficulty.” (Mother, case_MAL041S, Nyorro Jattaba)*

Within the homesteads paternal grandmothers were often elderly and mothers perceived their contribution to the care of infants as being mainly to carry them while the mothers undertook their domestic chores. Their role in infant feeding and rearing therefore appeared to be marginal with mothers opting to use the recommended infant feeding information from health care workers (HCWs) in their decision-making processes.

Mothers also highlighted the importance of peer support in encouraging them to adhere to the recommended infant feeding and rearing strategies. Within villages the mothers met regularly and shared their knowledge and experiences of infant feeding with peer counsellors who had been trained by the Gambia National Nutrition Agency (NaNA) to promote exclusive breastfeeding under 6 months.

*“Yes, I spread the information among the women at our “bantabato” (meeting ground) to tell the women that we should all do it that way. ... I myself get up and ask them to give me my child to breastfeed.” (Mother, control_MAL256C, Jali)*

Peer support also proved critical when a mother was bereaved and therefore not able to care for her infant and young children. In these situations, her peers would take on the care of the children until she was able to herself but duration of this support was variable and appeared to depend on a mother’s ability to maintain good social interactions with her peers.

### Infant feeding difficulties

Whenever mothers experienced challenges with infant feeding they tried to find solutions amongst themselves and often did not consider consulting with a HCW. These challenges would only become evident to the HCWs when the infants were admitted to the MRC clinic with an acute illness or severe wasting. Having an infant who persistently cried even after being breastfed was perceived by mothers and the community at large as a sign of a “hungry baby” and mothers therefore opted to introduce pap even in the first week of life, as a breast milk supplement. Both primiparous and multiparous mothers often perceived breastmilk insufficiency. Multiparous mothers reported having adequate amounts of breast milk for the first few children but subsequently inadequate amounts to breastfeed the later infants. In addition, on the rare occasion when they were consulted, HCWs were not able to offer practical solutions to this challenge, but instead reassured carers that this was the norm for some women.

*“When he was a baby, N (mother) did not have milk. That disturbs her and it makes her to prepare food for him early, which can make him stop crying. …When we started cooking food for him, he stopped crying. When he eats until belly full, then he keeps quiet and sleeps for sometimes lying without disturbing anybody on anything.” (Father, case_MAL035Z, Jiffarong)*

Some infants refused to take pap and in food insecure households, mothers had no alternatives to offer so opted to increase the amount of breast milk in their diet, including those over 6 months of age. In adequately resourced households, mothers tried alternatives such as ground rice or potato powder, which some infants preferred and were therefore able to receive adequate volumes of complementary feeds. In addition, these infants were “force fed” by mothers who were desperate to ensure that they were adequately fed.

*“…Then when I cook and was giving to him, he doesn’t agree to drink. No matter how I force him, he takes it out. Now I cook porridge for him and later stop cooking it because he doesn’t want to drink it. I only give him breast milk.” (Mother, case_MAL065Y, Karantaba)*

### Maternal psychological stressors

Mothers in this community faced several adverse events that had an impact on their psychological status, hence limiting their ability to appropriately care for their infants and to access health promotion services. Religion was the basis for many of their coping strategies and HCW were also important in this process.

#### Ill health of child

Having a sickly infant who required several admissions to health care facilities was stressful for mothers. The admissions particularly when the infant was unwell were not conducive for health promotion as HCWs focused on treating and saving the infant’s life during which time the mothers were distressed and preoccupied with their infant’s illness. By contrast mothers whose infants were healthy could access and assimilate health promotion messages regularly.

#### Death

The death of a child was a common experience for mothers. They found it more difficult to recover from the death of an older child who they had formed a relationship with, than from the death particularly of a newborn infant. Their experience of miscarriages was secretive and often not shared with their husbands. However, although distressing, mothers showed resilience by rationalising these losses and therefore did not feel that these bereavements affected their parenting capacity and therefore the growth and nutrition of their infants.

*“When a human being dies, and now moves from you, it makes you sad. But it is God that created death. And also in terms of Islam, if you want to do it in another way, it diminishes your Islam. I was very used to him (deceased child) truly speaking when he was with me. When he was taken from me, I felt that someone was taken from me but I then held onto this that it is God who gave me and when He was giving to me I was not aware of it. Therefore, now if He is in need of him and I can take it to be God’s property.” (Mother, case_ MAL015R_Jali)*

In this community, widows had limited autonomy in decision making for themselves or their children. In addition to grieving for the loss of their husband, the cultural practice that required them to remarry a male relative of the deceased (levirate marriage) sometimes exposed them and their children to adverse psychosocial circumstances as they were often remarried as the third or fourth wife to husbands who did not have the resources to support them adequately. This in turn limited a mother’s ability to care for her infant optimally.

#### Lack of autonomy in child spacing

Although the norm in this community was for mothers to breast feed children until 2 years of age, short birth spacing reduced the length of time a mother could feed her index infant as the belief in the population is that pregnant women should not breastfeed. Mothers found this stressful as it constrained their ability to adhere to the societal norms of breastfeeding whilst increasing their work load i.e. increasing number of young children to care for.

*“… because you may have a child less than 2 years and you later have another one who is less than 1 year you then conceive another one. Now if you want to take care of them, one is just removed from breastfeeding, the other is lactating and pregnant with another. That caring becomes difficult.” (Mother, control_MAL138F, Jiffarong)*

## Discussion

Our study showed that an inappropriate complementary feeding was associated with severe wasting in infants. In addition, maternal factors that hindered adherence to the recommended infant feeding and rearing practices were lack of social support networks; psychosocial stressors and infant feeding difficulties in the context of a heavy burden of domestic chores. We also found some weak evidence of a protective effect of household water treatment on severe wasting that may suggest mothers’ abilities to prioritise domestic tasks within a supportive household or community. However, we found no evidence of the association between maternal depressive symptoms and severe wasting in infants in this population.

The association between suboptimal infant feeding practices and severe wasting has previously been reported this population [25]. On further exploration of factors associated with the high frequency of complementary feeds in our study, we found that mothers were often unable to supervise infant feeding sessions, due to the burden of domestic chores. As a result, infants were likely to have ingested inadequate quantities of food necessitating more regular feeds. These findings are consistent with a report of time allocation for rural African women that estimates they spend 3 h a day on meal preparation and only 1 hour for child care [57]. However, the increased odds of wasting associated with the increased frequency of feeds in infants may also be explained by the tendency of mothers of severely wasted infants to increase the frequency of feeds sometimes to over five times a day. There is a general expectation that increasing the frequency of feeding should improve the nutritional status of infants particularly after an episode of diarrhoea [58]. This practice may have diminishing returns as often the total daily amount consumed by infants does not increase beyond 4 or 5 meals per day [59].

We found that mothers who experienced food insecurity and were unable to diversify their infants’ diets, opted to continue exclusive breastfeeding well beyond the upper recommended limit of 6 months. This exposed their infants to significant deficits in their energy and nutrient intake at a critical time in their growth and development, with the immediate risk of severe wasting and adverse long-term nutritional and developmental outcomes [60]. These findings highlight the need for more targeted complementary feeding education messages and strategies that address the specific challenges that rural African mothers face such as food insecurity. HCWs in LMICs should also be trained and supported to employ communication strategies that foster discussion with carers about the problems that they face in infant feeding and rearing, in order to identify and address them in a timely manner before the onset of growth faltering.

The overall prevalence of maternal depressive symptoms was 13%, which was similar to the prevalence reported in a neighbouring rural Gambian community [41]. This finding was not unexpected in the context of maternal sequential or concurrent exposure to psychosocial stressors including poverty, food insecurity, marital discord, death & ill health of their infants or husbands and a heavy workload in both the antenatal and postnatal periods. However, maternal depressive symptoms were not significantly associated with severe wasting in infancy in our study. This could partly be explained by the fact that mothers rarely reported feelings of low mood or inability to cope with their daily lives, even in the context of adverse events. We interpreted this as a coping strategy in the context of societal expectations for mothers to demonstrate resilience even in the face of adversity. In addition, we observed that adequate support networks were key to promoting resilience in mothers and their infants living under adverse psychosocial conditions, as has been reported before in this community [29]. The support of their husbands was also critical to this process not only for financial, but also for emotional support to the family. Historically, most child nutrition interventions have focused on maternal involvement [15]. However, there is growing recognition that the role of the father is vital in the growth and development of children and is not limited to the provision of resources [61, 62]. The evolving roles of fathers even in LMICs as partners in child care [63], presents opportunities to involve them in the counselling of infant feeding and other child care practices that promote infant and child growth and development.

Encouragingly we found that the younger fathers in our study acknowledged and were keen to engage in their evolving roles as fathers, and were keen to be learn and support mothers in child care. This is a theme that needs to be explored further.

The support from the maternal grandmothers was also key to supporting infant feeding through provision of resources when mothers faced food insecurity. Although mothers often lived in the same compound as the paternal grandmothers, their role in child care was often limited to carrying the infants while their mothers attended to their domestic or farming chores as they were often frail. Previous work in this rural Gambian community showed a clear beneficial effect of maternal grandmothers on both child growth and survival but not with paternal grandmother [28]. Peer support was also important in giving practical support with child care when mothers were incapacitated due to bereavement or illness. Peers were also a useful source of advice on infant feeding strategies particularly if they had been trained as peer supporters. The role of peer support groups within LMIC communities has been shown to result in sustained improvements in infant feeding practices [64].

### Strengths and limitations

A key strength of this mixed methods study approach is that it enabled us to investigate infant feeding and rearing practises in great depth and explore the complex phenomena of the pathways to severe wasting in infants in this rural Gambian community. In addition, our iterative study design enabled us to visit the homes of the respondents 2–3 times from the formative phase to the interview phase. This helped us to build a rapport with the carers the key household members, which facilitated the sharing of sensitive information around family dynamics and infant rearing. It also allowed us to check whether initial responses were captured accurately. This prolonged engagement with participants enhances the credibility and dependability of findings in qualitative studies [65].

A main limitation of this study is that data on many of the exposures were collected retrospectively, and often over 12 months after the child had growth faltered with the associated challenges of recall bias for example, the carers of cases may have been able to recall information on the exposures of interest in greater detail. Alternatively, having a severely malnourished child may have resulted in carers exaggerating certain aspects of care for example the timing and frequency of complementary feeds. We have attempted to mitigate these influences by using the prospectively collected data where it was available, as actual exposure variables (complementary feeding, wealth indicators, infant morbidity, parity). In addition, the inability of this study to capture many known risk factors of severe wasting may be because the minimum calculated sample size for this study may have led to a lack of power to detect statistically significant difference. However, the findings from the mixed methods approach still provide useful information that can guide the development of strategies to prevent wasting in infants in this rural Gambian community.

A limitation of qualitative research is that it is context-specific and therefore the findings are not generalizable to other contexts [66]. The findings of this study in one rural district of The Gambia may therefore not be applicable to other settings within The Gambia or the West African sub-region. However, the themes raised can be explored in other settings. The findings can also be used to generate theory that can be explored in other rural African settings with similar cultural and socioeconomic backgrounds.

## Conclusion

In rural Gambia, mothers were the primary caregivers of infants and were often the recipients of infant feeding advice from health care providers. However, adherence to the recommended infant feeding and rearing strategies was constrained by their adverse psychosocial circumstances. In addition, inappropriate complementary feeding practices were associated with severe wasting in infants. Future strategies to prevent severe wasting in infants in this community and similar low resource settings should incorporate interventions that promote maternal resilience including: gender empowerment, maternal psychosocial support, and foster the involvement of fathers in infant and child health and nutrition promotion.

## Additional files

Additional file 1: additional information on methods, results, tables figures and references (DOCX 91 kb) (DOCX 87 kb)

Additional file 2: Tool to assess infant feeding, socioeconomic status and water/hygiene/sanitation conditions (PDF 291 kb)

Additional file 3: Tool for assessment of maternal mental health (PDF 226 kb)

Additional file 4: Tool to support interviews with mothers (DOCX 68 kb)

Additional file 5: Tool for to support interviews with fathers (DOCX 67 kb)

## Abbreviations

CMDs: Common Mental Disorders
EDS: Edinburgh Depression Scale
FGD: Focus group discussion
HAZ: Height-for-age z-score
HCW: Health care worker
IDI: In-depth interview
IQR: Interquartile range
LMIC: Low and middle income countries
MRC: Medical Research Council
NaNA: The Gambia National Nutrition Agency
PCA: Principal Component Analysis
RF: Responsive feeding
WASH: Water Hygiene and Sanitation
WAZ: Weight-for-age z-score
WLZ: Weight-for-length z-score

## References

[1] Black RE, Victora CG, Walker SP, Bhutta ZA, Christian P, de Onis M, Ezzati M (2013). Grantham-McGregor S, Katz J, Martorell R *et al*: maternal and child undernutrition and overweight in low-income and middle-income countries. Lancet.

[2] Levels and trends in child malnutrition, UNICEF-WHO-The World Bank joint child malnutrition estimates [http://data.unicef.org/wp-content/uploads/2016/09/UNICEF-Joint-Malnutrition-brochure.pdf (Accessed 5 Jan 2017)].

[3] Victora CG, Adair L, Fall C, Hallal PC, Martorell R, Richter L, Sachdev HS (2008). Maternal, child Undernutrition study G: maternal and child undernutrition: consequences for adult health and human capital. Lancet.

[4] Galler J, Rabinowitz DG (2014). The intergenerational effects of early adversity. Prog Mol Biol Transl Sci.

[5] Lelijveld N, Seal A, Wells JC, Kirkby J, Opondo C, Chimwezi E, Bunn J, Bandsma R, Heyderman RS, Nyirenda MJ (2016). Chronic disease outcomes after severe acute malnutrition in Malawian children (ChroSAM): a cohort study. Lancet Glob Health.

[6] Rubin LP (2016). Maternal and pediatric health and disease: integrating biopsychosocial models and epigenetics. Pediatr Res.

[7] Joint Malnutrition dataset from UNICEF, World Bank and WHO [http://data.unicef.org/wp-content/uploads/2015/12/JME_December_2016.xlsx]. Accessed 18 Dec 2017.

[8] Rivera J, Ruel MT (1997). Growth retardation starts in the first three months of life among rural Guatemalan children. Eur J Clin Nutr.

[9] Schwinger C, Fadnes LT, Shrestha SK, Shrestha PS, Chandyo RK, Shrestha B, Ulak M, Bodhidatta L, Mason C, Strand TA (2016). Predicting Undernutrition at age 2 years with early attained weight and length compared with weight and length velocity. J Pediatr.

[10] Prentice AM, Moore SE, Fulford AJ (2013). Growth faltering in low-income countries. World Rev Nutr Diet.

[11] UNICEF (2015). European Union: multi-sectorial approaches to nutrition: nutrition-specific and nutrition sensitive interventions to accelerate progress in.

[12] Tette EM, Sifah EK, Nartey ET, Nuro-Ameyaw P, Tete-Donkor P, Biritwum RB (2016). Maternal profiles and social determinants of malnutrition and the MDGs: what have we learnt?. BMC Public Health.

[13] Ashaba S, Rukundo GZ, Beinempaka F, Ntaro M, LeBlanc JC (2015). Maternal depression and malnutrition in children in southwest Uganda: a case control study. BMC Public Health.

[14] Bhutta ZA, Ahmed T, Black RE, Cousens S, Dewey K, Giugliani E, Haider BA, Kirkwood B, Morris SS, Sachdev HP (2008). What works? Interventions for maternal and child undernutrition and survival. Lancet.

[15] Bhutta ZA, Das JK, Rizvi A, Gaffey MF, Walker N, Horton S, Webb P, Lartey A, Black RE (2013). Lancet nutrition interventions review G *et al*: evidence-based interventions for improvement of maternal and child nutrition: what can be done and at what cost?. Lancet.

[16] Bhutta ZA (2013). Early nutrition and adult outcomes: pieces of the puzzle. Lancet.

[17] Ruel MT, Alderman H, Maternal, Child Nutrition Study G (2013). Nutrition-sensitive interventions and programmes: how can they help to accelerate progress in improving maternal and child nutrition?. Lancet.

[18] Stewart RC (2007). Maternal depression and infant growth: a review of recent evidence. Maternal & child nutrition.

[19] Surkan PJ, Patel SA, Rahman A (2016). Preventing infant and child morbidity and mortality due to maternal depression. Best practice & research Clinical obstetrics & gynaecology.

[20] Adewuya AO, Ola BO, Aloba OO, Mapayi BM, Okeniyi JA (2008). Impact of postnatal depression on infants' growth in Nigeria. J Affect Disord.

[21] Harpham T, Huttly S, De Silva MJ, Abramsky T (2005). Maternal mental health and child nutritional status in four developing countries. J Epidemiol Community Health.

[22] Surkan PJ, Kennedy CE, Hurley KM, Black MM (2011). Maternal depression and early childhood growth in developing countries: systematic review and meta-analysis. Bull World Health Organ.

[23] The Gambia Bureau of Statistics (GBOS), UNICEF (2011). The Gambia- multiple indicator cluster survey 2010.

[24] Nabwera HM, Fulford AJ, Moore SE, Prentice AM (2017). Growth faltering in rural Gambian children after four decades of interventions: a retrospective cohort study. Lancet Glob Health.

[25] Hoare K (1994). Tackling infant malnutrition in the Gambia. Health visitor.

[26] Thomas JE, Dale A, Bunn JE, Harding M, Coward WA, Cole TJ, Weaver LT (2004). Early helicobacter pylori colonisation: the association with growth faltering in the Gambia. Arch Dis Child.

[27] Campbell DI, Elia M, Lunn PG (2003). Growth faltering in rural Gambian infants is associated with impaired small intestinal barrier function, leading to endotoxemia and systemic inflammation. J Nutr.

[28] Sear R, Mace R, McGregor IA (2000). Maternal grandmothers improve nutritional status and survival of children in rural Gambia. Proceedings Biological sciences/The Royal Society.

[29] Mwangome M, Prentice A, Plugge E, Nweneka C (2010). Determinants of appropriate child health and nutrition practices among women in rural Gambia. J Health Popul Nutr.

[30] Moore SE, Fulford AJ, Darboe MK, Jobarteh ML, Jarjou LM, Prentice AM (2012). A randomized trial to investigate the effects of pre-natal and infant nutritional supplementation on infant immune development in rural Gambia: the ENID trial: early nutrition and immune development. BMC pregnancy and childbirth.

[31] Creswell JW (2009). Research design- qualitative, quantitative and mixed methods.

[32] Hennig BJ, Unger SA, Dondeh BL, Hassan J, Hawkesworth S, Jarjou L, Jones KS, Moore SE, Nabwera HM, Ngum M (2015). Cohort profile: the kiang west longitudinal population study (KWLPS)-a platform for integrated research and health care provision in rural Gambia. Int J Epidemiol.

[33] Countrystat website Gambia [http://www.countrystat.org/home.aspx?c=GMB&p=ke ]. Accessed 18 Dec 2017.

[34] UN Systems, The Gambia (2012). The Gambia response and recovery plan for 2013: addressing the emergency and investing in resilience.

[35] Rayco-Solon P, Moore SE, Fulford AJ, Prentice AM (2004). Fifty-year mortality trends in three rural African villages. Tropical medicine & international health : TM & IH.

[36] Eriksen KG, Johnson W, Sonko B, Prentice AM, Darboe MK, Moore SE (2016). Following the World Health Organization's recommendation of exclusive breastfeeding to 6 months of age does not impact the growth of rural Gambian infants. J Nutr.

[37] The WHO Growth Reference Standards [http://www.who.int/childgrowth/en/]. Accessed 18 Dec 2017.

[38] Johnson W, Darboe MK, Sosseh F, Nshe P, Prentice AM, Moore SE (2016). Association of prenatal lipid-based nutritional supplementation with fetal growth in rural Gambia. Maternal & child nutrition.

[39] Patton MQ (1990). Qualitative evaluation and research methods.

[40] Guest G, Bunce A, Johnson L (2006). How many interviews are enough? An experiment with data saturation and variability. Field Methods.

[41] Coleman R, Morison L, Paine K, Powell RA, Walraven G (2006). Women's reproductive health and depression: a community survey in the Gambia, West Africa. Soc Psychiatry Psychiatr Epidemiol.

[42] Cox JL, Chapman G, Murray D, Jones P (1996). Validation of the Edinburgh postnatal depression scale (EPDS) in non-postnatal women. J Affect Disord.

[43] Ali GC, Ryan G, De Silva MJ (2016). Validated screening tools for common mental disorders in low and middle income countries: a systematic review. PLoS One.

[44] Process of translation and adaptation of instruments [http://www.who.int/substance_abuse/research_tools/translation/en/]. Accessed 18 Dec 2017.

[45] Pope C, Mays N (2006). Qualitative research in health care.

[46] Kvale S (1996). An introduction to qualitative research: SAGE.

[47] Kaiser HF (1974). An index of factorial simplicity. Psychometrika.

[48] Filmer D, Pritchett LH (2001). Estimating wealth effects without expenditure data--or tears: an application to educational enrollments in states of India. Demography.

[49] Kirkwood BR, JAC S, Kirkwood BR, JAC S (2008). Regression modelling. Essential Medical Statistics.

[50] Creswell JW (2012). Qualitative inquiry and research design: choosing among five approaches.

[51] Pope C, Ziebland S, Mays N (2000). Qualitative research in health care. Analysing qualitative data. BMJ.

[52] Tuckett AG (2005). Applying thematic analysis theory to practice: a researcher's experience. Contemp Nurse.

[53] Carey JW, Morgan M, Oxtoby MJ (1996). Intercoder agreement in analysis of responses to open-ended interview questions: examples from tuberculosis research. Cultural Anthropology Methods.

[54] Braun V, Clarke V (2006). Using thematic analysis in psychology. Qual Res Psychol.

[55] Braun V, Clarke V (2014). What can "thematic analysis" offer health and wellbeing researchers?. International journal of qualitative studies on health and well-being.

[56] Pope C, Ziebland S, Mays N, Pope C, Ziebland S, Mays N (2006). Analysing qualitative data, thematic analysis. Qualitiative research in health care.

[57] McGuire JS, Austin JE (1987). Children's growth for national development.

[58] Paintal K, Aguayo VM (2016). Feeding practices for infants and young children during and after common illness. Evidence from South Asia. Maternal & child nutrition.

[59] Brown KH, Creed-Kanashiro, Dewey K (1995). Optimal complementary feeding practices to prevent childhood malnutrition in developing countries. Food Nutr Bull.

[60] Dewey KG, Adu-Afarwuah S (2008). Systematic review of the efficacy and effectiveness of complementary feeding interventions in developing countries. Maternal & child nutrition.

[61] Grantham-McGregor SM, Pollitt E, Wachs TD, Meisels SJ, Scott KG (1999). Summary of the scientific evidence on the nature and determinants of child development and their implications for programmatic interventions with young children. Food Nutr Bull.

[62] Engle PL, Pelto G, Bentley P (2000). Care for nutrition and development. J Indian Med Assoc.

[63] Lamb ME (2000). The history of research on father involvment: an overview. Marriage Fam Rev.

[64] Kushwaha KP, Sankar J, Sankar MJ, Gupta A, Dadhich JP, Gupta YP, Bhatt GC, Ansari DA, Sharma B (2014). Effect of peer counselling by mother support groups on infant and young child feeding practices: the Lalitpur experience. PLoS One.

[65] Krefting L (1991). Rigor in qualitative research: the assessment of trustworthiness. The American journal of occupational therapy : official publication of the American Occupational Therapy Association.

[66] Al-Busaidi ZQ (2008). Qualitative research and its uses in health care. Sultan Qaboos Univ Med J.

