# Supplementary material for: The influence of maternal psychosocial circumstances and physical environment on the risk of severe wasting in rural Gambian infants: a mixed methods approach
Source: BMC Public Health. 2018 Jan 6;18:109. doi: 10.1186/s12889-017-4984-2 (PMC5756408; doi:10.1186/s12889-017-4984-2)
Supplement: Supplementary file 1 — additional information on methods, results, tables figures and references (DOCX 91 kb) (DOCX 87 kb) [file 12889_2017_4984_MOESM1_ESM.docx]

**The influence of maternal psychosocial circumstances and physical environment on the risk of severe wasting in rural Gambian infants: a mixed methods approach**

Nabwera HM, ^1,2^ Moore SE,^3^ Mwangome MK,^4^ Molyneux CS,^4,5^ Darboe MK,^1^ Camara-Trawally N,^1^ Sonko B,^1^ Darboe A^1^, Singhateh S^1^, Fulford AJ,^1,2^ Prentice AM^1,2^

1. Medical Research Council Unit, The Gambia, P. O. Box 273, Banjul, The Gambia
2. Department of Population Health, London School of Hygiene and Tropical Medicine, Keppel street, London, WC1E 7HT, United Kingdom
3. Division of Women’s Health, King’s College London, 10th floor North Wing, St Thomas’ Hospital, Westminster Bridge Road, London, SE1 7EH, United Kingdom
4. Kenya Medical Research Institute-Wellcome Trust Research Programme, P.O.Box 230-80108, Kilifi, Kenya
5. University of Oxford, Nuffield Department of Medicine, Henry Wellcome Building for Molecular Physiology, Old Road Campus, Headington, Oxford OX3 7BN

## Supplementary material- contents

1. Supplementary methods
2. Supplementary results
3. Supplementary tables
4. Supplementary figures
5. Supplementary references

### Supplementary methods

#### Study population

The Early Nutrition and Immune Development Trial (ENID, ISRCTN49285450) is a randomised trial designed to investigate the effects of combined pre-natal and infancy nutritional supplements on infant immune development. In the pre-natal arm, pregnant women (from <20weeks gestation) were randomized to 4 intervention arms [[1](#_ENREF_1)]. From 6 months of age All pregnant women underwent voluntary counselling and testing for HIV as part of routine antenatal care and those found to be HIV infected were not recruited into the ENID trial, but were referred to the closest HIV care facility [[2](#_ENREF_2)]. In total 875 mother-infant pairs were recruited into this trial from 2010-2015 [[1](#_ENREF_1)].

**Setting**

Polygamy is a popular and acceptable practice and isolated nuclear family units are rare as most compounds will have extended family relations including grandparents living within the family unit. In rural Gambia, the value and status of women is often based on their reproductive capacity therefore fertility rates have remained high and the uptake of contraception low [[3](#_ENREF_3)]. The majority of married women live in a compound belonging to their husband or his family [[4](#_ENREF_4)].

**Data collection**

***Quantitative***

***Mental Health Questionnaire***

The Edinburgh Depression Scale (EDS) consists of ten questions and a woman can rate her depression symptoms on a scale of 0 (none) to 3 (severe). The total score ranges from 0 to 30 and scores of ≥12 are suggestive of depression. The reported sensitivity is 79% and specificity 85% [[5](#_ENREF_5), [6](#_ENREF_6)]. We utilised the principles of the WHO translation protocol with “emphasis on the conceptual and cultural equivalence and not on the linguistic equivalence” [[7](#_ENREF_7)].

**Data analysis**

**Quantitative**

**Principal component analysis**

For the PCA, all the sociodemographic variables were converted to binary variables where missing values of distinct binary variables were replaced by the means of all summarized "0" values (asset not present) and "1" values (asset present). Initially all the variables that were based on the 10 indicators of childhood poverty as stated in the multi-dimensional poverty index report [[8](#_ENREF_8)], were added to the PCA. This generated 30 principal components. Using the Kaiser-Meyer-Olkin (KMO) measure of sampling adequacy [[9](#_ENREF_9)], variables with a KMO value of <0.50 or missing values were dropped from the model. The final PCA had 6 variables that were measures of assets, education and distance from the drinking water sources (Table 3). The overall value was 0.62 (mediocre) (Table 4). The first two principal components where the associated eigenvalue was greater than one were selected assuming that the first principal component was a measure of economic status (Figure 1).[[10](#_ENREF_10), [11](#_ENREF_11)] “The eigenvalue (variance) for each principal component indicates the percentage of variation in the total data explained”[[11](#_ENREF_11)].

## Supplementary results

*Characteristics of cases in the first 12 months*

The mean age of first growth faltering episode when WLZ< -3 was 6.5 (SD 3.5) months and at 12 months their mean WLZ was -2.07 (SD 1.32, n=53). There were more male infants (48 [57.8%]) among the cases. The mean maternal and paternal ages were 34.9 (SD 6.37) and 49.2 (SD 12.4) years respectively. The largest proportion of infants were from a medium and a large sized village that were 5km and 19km from the MRC clinic (Table 6).

*Maternal*

During the study only one participant who was found to have significant depressive symptoms did not respond to these primary care interventions and was referred to the Edward Francis Small Teaching Hospital in Banjul for specialist psychiatric assessment and management.

## Supplementary table

## Table S1: Inclusion and exclusion criteria for the ENID randomized trial[[1](#_ENREF_1)]

| Inclusion criteria for ENID trial | | Exclusion criteria for ENID trial | |
| --- | --- | --- | --- |
| Women | Infants | Women | Infants |
| Resident in West Kiang and aged between 18 – 45 y at August 1^st^ 2008 | All infants born to women enrolled into the pre-natal arm of the study | Currently enrolled in another MRC study | Major congenital malformations |
| Planning to remain resident in West Kiang for the next 36 months |  |  |  |
|  |  | Current pregnancy (beyond 20 wk on ultrasound assessment) | **Severe malnutrition (weight-for-height Z-score < -3)** |
|  |  | Severe anaemia (haemoglobin < 7 g/dL) |  |
|  |  | Known sickle cell disease |  |
|  |  | Reported onset of menopause |  |
|  |  | Known HIV infected |  |

**Table S2: Sample size estimation**

|  |  | Number of cases -> | | | | |
| --- | --- | --- | --- | --- | --- | --- |
|  |  | **33** | **40** | **50** | **75** | **132** |
| prop. controls exposed | **0.1** | 5.81 | 5.10 | 4.43 | 3.50 | 2.66 |
|  | **0.2** | 4.18 | 3.72 | 3.29 | 2.70 | 2.15 |
|  | **0.3** | 3.69 | 3.31 | 2.95 | 2.45 | 1.98 |
|  | **0.4** | 3.55 | 3.18 | 2.83 | 2.36 | 1.92 |
|  | **0.5** | 3.62 | 3.23 | 2.85 | 2.36 | 1.91 |
|  | **0.6** | 3.93 | 3.45 | 3.02 | 2.45 | 1.96 |
|  | **0.7** | 4.68 | 4.00 | 3.41 | 2.69 | 2.09 |
|  | **0.8** | 7.06 | 5.61 | 4.49 | 3.28 | 2.39 |
|  | **0.9** | 128.24 | 23.84 | 11.88 | 6.01 | 3.48 |

Alpha 5%; Beta 90%; Controls per case 3

**Table S3: Eigenvectors in final principal component analysis model**

| Variable | Comp1 | Comp2 | Comp3 | Comp4 | Comp5 | Comp6 |
| --- | --- | --- | --- | --- | --- | --- |
| Electricity | 0.58 | -0.24 | -0.12 | -0.20 | 0.14 | -0.73 |
| TV | 0.55 | -0.22 | -0.03 | -0.24 | -0.26 | 0.67 |
| Cart | 0.26 | 0.61 | -0.24 | -0.33 | -0.62 | 0.01 |
| Bicycle | 0.20 | 0.68 | -0.10 | 0.41 | 0.57 | -0.06 |
| Motorcycle | 0.46 | -0.16 | 0.24 | 0.71 | -0.43 | 0.09 |
| Car | 0.22 | 0.19 | 0.88 | -0.34 | 0.12 | 0.08 |

**Table S4: Kaiser-Meyer-Olkin measure of sampling adequacy of final principal component analysis model**

| Variable | KMO* measure |
| --- | --- |
| Motorcycle | 0.77 |
| Car | 0.62 |
| TV | 0.60 |
| Electricity | 0.59 |
| Cart | 0.57 |
| Bicycle | 0.52 |
| Overall | 0.61 |

*Kaiser-Meyer-Olkin

**Table S5: Principal component analysis 2 comparison between cases and controls**

| Principal component analysis 1 | Cases N=77 | Control N=203 | P value |
| --- | --- | --- | --- |
| Wealth quintiles, n (%)  1 (Poorest)  2  3  4  5 (Wealthiest) | 34 (44)  2 (3)  26 (34)  3 (4)  12 (16) | 74 (37)  3 (2)  72 (35)  11 (5)  43 (21) | 0.15* |

*Wilcoxon Ranksum test

**Table S6:** **Characteristics of cases during first 12 months of life**

| Characteristics | | | N=77 |
| --- | --- | --- | --- |
| Age at first WHZ <-3, mean (SD) | | | 6.5 (3.6) |
| Age of introduction complementary foods, mean (SD) (prospective) | | | 5.2 (1.2)) |
|  | | |  |
| Village of residence  Keneba  Kantong Kunda  Manduar  Tankular  Kuli Kunda  Joli  Bajana  Karantaba  Jiffarong  Burong  Sankandi  Nyorro Jattaba  Jattaba  Kemoto  Dumbuto  Batelling | **Village size**  Large  Medium  Medium  Medium  Large  Medium  Medium  Large  Large  Medium  Medium  Large  Large  Medium  Medium  Small | **Distance from MRC clinic in km**  0  5  8  10  12  13  16  17  19  21  23  24  26  27  30  42 | **N (%)**  4 (5)  12 (15)  2 (3)  6 (8)  8 (10)  5 (6)  2 (3)  1 (1)  12 (15)  4 (5)  3 (4)  6 (8)  5 (6)  5 (6)  2 (3)  2 (3) |

## Supplementary figures

**Figure 1: Scree plot of eigenvalues from principal component analysis**

**Figure 2: UNICEF conceptual framework for undernutrition (adapted) [**[**12**](#_ENREF_12)**]**

Intergenerational consequences

Long-term consequences

(cognitive development, health,

Economic productivity)

Short-term consequences

(mortality, morbidity, disability)

Maternal and Child undernutrition

Disease

Inadequate dietary intake

**IMMEDIATE CAUSES**

**UNDERLYING**

Household food insecurity

Inadequate foods, feeding and care practices

House, environment and health services

**CAUSES**

**BASIC CAUSES**

Inadequate access to services

Inadequate financial and human resources

Sociocultural , economic and political context

## Supplementary references

1. Moore SE, Fulford AJ, Darboe MK, Jobarteh ML, Jarjou LM, Prentice AM: **A randomized trial to investigate the effects of pre-natal and infant nutritional supplementation on infant immune development in rural Gambia: the ENID trial: Early Nutrition and Immune Development**. *BMC pregnancy and childbirth* 2012, **12**:107.

2. Johnson W, Darboe MK, Sosseh F, Nshe P, Prentice AM, Moore SE: **Association of prenatal lipid-based nutritional supplementation with fetal growth in rural Gambia**. *Maternal & child nutrition* 2016.

3. Bledsoe CH: **Contingent Lives: Fertility, Time, and Aging in West Africa**. In*.* Chicago; 2002.

4. Cassell JA, Leach M, Fairhead JR, Small M, Mercer CH: **The social shaping of childhood vaccination practice in rural and urban Gambia**. *Health policy and planning* 2006, **21**(5):373-391.

5. Cox JL, Chapman G, Murray D, Jones P: **Validation of the Edinburgh Postnatal Depression Scale (EPDS) in non-postnatal women**. *Journal of affective disorders* 1996, **39**(3):185-189.

6. Ali GC, Ryan G, De Silva MJ: **Validated Screening Tools for Common Mental Disorders in Low and Middle Income Countries: A Systematic Review**. *PloS one* 2016, **11**(6):e0156939.

7. **Process of translation and adaptation of instruments** [<http://www.who.int/substance_abuse/research_tools/translation/en/>]

8. Santos ME, Alkire S: **The multidimensional poverty index**. In*.*; 2011.

9. Kaiser HF: **An index of factorial simplicity**. *Psychometrika* 1974, **39**:31-36.

10. Houweling TA, Kunst AE, Mackenbach JP: **Measuring health inequality among children in developing countries: does the choice of the indicator of economic status matter?** *International journal for equity in health* 2003, **2**(1):8.

11. Vyas S, Kumaranayake L: **Constructing socio-economic status indices: how to use principal components analysis**. *Health policy and planning* 2006, **21**(6):459-468.

12. UNICEF, European Union: **Multi-sectorial approaches to nutrition: nutrition-specific and nutrition sensitive interventions to accelerate progress** In*.*; 2015.
