# Supplementary material for: The influence of maternal psychosocial circumstances and physical environment on the risk of severe wasting in rural Gambian infants: a mixed methods approach
Source: BMC Public Health. 2018 Jan 6;18:109. doi: 10.1186/s12889-017-4984-2 (PMC5756408; doi:10.1186/s12889-017-4984-2)
Supplement: Supplementary file 2 — Tool to assess infant feeding, socioeconomic status and water/hygiene/sanitation conditions (PDF 291 kb) [file 12889_2017_4984_MOESM2_ESM.pdf]

Medical Research Council Unit, The Gambia

Evaluation of the Risk Factors for Malnutrition in Children Recruited to a Supplementation Trial in Rural Gambia

MRC | The Gambia Unit

QUESTIONNAIRE

Version 03, 15th Sept. 2014

WestKiang No: #Name? Date of Birth: #Name? Study ID:

Visit date: |__|__|/|__|__|/|__|__|__|__| Fieldworker Name:

Section 1: Demographic and Socio-economic Information

1. Who is the child's current primary carer?

☐ Mother [01] ☐ Father [02] ☐ Brother [03] ☐ Sister [04] ☐ Maternal Grandmother [05]
☐ Paternal Grandmother [06] ☐ Maternal Grandfather [07] ☐ Paternal Grandfather [08] ☐ Maternal Auntie [09] ☐ Paternal Auntie [10]
☐ Maternal Uncle [11] ☐ Paternal Uncle [12] ☐ Co-wife [13] ☐ No Relation [14] ☐ Other Relation [15]

2. Who was the child's primary carer between 0-12 months of age?

☐ Mother [01] ☐ Father [02] ☐ Brother [03] ☐ Sister [04] ☐ Maternal Grandmother [05]
☐ Paternal Grandmother [06] ☐ Maternal Grandfather [07] ☐ Paternal Grandfather [08] ☐ Maternal Auntie [09] ☐ Paternal Auntie [10]
☐ Maternal Uncle [11] ☐ Paternal Uncle [12] ☐ Co-wife [13] ☐ No Relation [14] ☐ Other Relation [15]

3. Where does the child's mother live?

☐ In household [1] ☐ Outside of household within the Gambia [2] ☐ Abroad [3] ☐ Whereabouts unknown [4] ☐ Died [5]

4. Where does the child's father live?

☐ In household [1] ☐ Outside of household within the Gambia [2] ☐ Abroad [3] ☐ Whereabouts unknown [4] ☐ Died [5]

5. How many children younger than 5 years live in the household? |___|___|

6. Are all the child's siblings alive? If not, please give the estimated age and cause of death of the deceased siblings. ☐ Yes [1] ☐ No [0]

	Age of Death	Cause of Death
1		
2		
3		
4		
5		
6		

	Age of Death	Cause of Death
7		
8		
9		
10		
11		
12		

7. What level of formal education has the primary carer had?

☐ No formal education [1] ☐ Arabic School [2] ☐ Less than primary school [3] ☐ Completed primary school [4]
☐ Completed secondary school [5] ☐ Post-secondary school [6] ☐ Don't know [7]

8. What is the carer's main source of income?

☐ Farming [1] ☐ Business [2] ☐ Salary [3]
☐ Trading [4] ☐ Remuneration from children [5] ☐ Other, specify ________________________________ [6]

9. Is the above income guaranteed every month? ☐ Yes [1] ☐ No [0]

10. How many rooms in your household are used for sleeping? |___|___|

11. How many people have been sleeping regularly in your household over the past 6 months? |___|___|

12. Does the mother/carer have the freedom to move around the community to access resources such as markets, shops, clinics, and companions...

☐ Alone [1]? ☐ With child [2]? ☐ with another same-aged woman [3]? ☐ Only with a husband or elder [4]? ☐ Never [5]?

13. Who decides what medical care to give the child?

☐ Mother alone [1] ☐ Jointly with husband [2] ☐ Jointly with mother-in-law [3] ☐ Jointly with another person [4]
☐ Mother has no say [5]

Section 2: Infant Feeding Practices

1. Is child currently breastfed? ☐ No [0] ☐ Partial breastfeeding [1] ☐ Exclusive breastfeeding [2]

2. If no, has the child ever been breast fed? ☐ Yes [1] ☐ No [0]

3. If yes, what type of breastfeeding was it? ☐ Partial breastfeeding [1] ☐ Exclusive breastfeeding [2

4. If Yes, what age was the child when breastfeeding was stopped? |___|___| months

5. If No, what feeds did the child receive soon after birth? ________________________________

6. At what age did you introduce complementary feeds? |___|___| months

7. What complementary feeds did the child receive before 12 months of age? ____________________

8. Please list the types of food that you give to the child regularly?

1		2		3		4		5	

9. Who decides what food to feed the child? ☐ Mother alone [1] ☐ Jointly with husband [2]
☐ Jointly with mother-in-law [3] ☐ Jointly with another person [4]
☐ Mother has no say [5]

10. Who feeds the child most of the time?

☐ Mother [01] ☐ Father [02] ☐ Brother [03] ☐ Sister [04] ☐ Maternal Grandmother [05]
☐ Paternal Grandmother [06] ☐ Maternal Grandfather [07] ☐ Paternal Grandfather [08] ☐ Maternal Auntie [09] ☐ Paternal Auntie [10]
☐ Maternal Uncle [11] ☐ Paternal Uncle [12] ☐ Co-wife [13] ☐ No Relation [14] ☐ Other Relation [15]

11. What do you use to feed the child? ☐ Hand [1] ☐ Spoon [2] ☐ Other, please specify________________ [3]

12. How many times a day is the child fed on complementary feeds? |___|___|

13. Regarding feeding times for the child: ☐ Scheduled [1] ☐ Fed when hungry [2]

14. How do you assess to see whether the child is hungry? ____________________________________

____

15. How do you assess to see whether the child is full? ____________________________________

____

16. At most feeding times: ☐ Own bowl [1] ☐ Share with other children [2] ☐ Share with adults [3]

Section 3: Water, Sanitation and Hygiene

1. Over the past 6 months, what was the main source of water for the members of your household

☐ Piped into house [1] ☐ Covered well in house or compund [2] ☐ Piped into yard [3] ☐ Covered public well [4]
☐ Public tap [5] ☐ Open public well River or stream [6] ☐ Open well in house or yard [7] ☐ Deep tube well [8]
☐ Rainwater [9] ☐ Shallow tube well [10] ☐ Bought (tank, bottles, etc) [11] ☐ Borehole [12]
☐ Other, please specify____ [13]

2. How long does it take to go there, get water, and come back?

☐ Less than 15 minutes [1] ☐ 15 - 29 minutes [2] ☐ 30 - 59 minutes [3] ☐ 1 - 3 hours [4] ☐ More than 3 hours [5]

3. Do you or other members from your household go and fetch drinking water for the household every day?

☐ Yes [1] ☐ No [0]

4. If yes, on average, how many trips do you and members from your household make to fetch water each day?

|___|___|

5. Do you usually treat drinking water at home?

☐ Yes [1] ☐ No [0]

6. If yes, which method do you use the most to treat drinking water at home? (Tick all that applies)

☐ Leave water in sun to disinfect [1] ☐ Boil [2] ☐ Filter through a cloth [3] ☐ Filter through ceramic or other filter [4]
☐ Chlorine liquid, powder, or tablets Alum [5] ☐ Other chemical or additive, specify____ [6]

7. If you use chlorine liquid, powder or tablets, which type do you most commonly use? (Tick only one)

☐ Certeza [1] ☐ Watermaker [2] ☐ Aquatabs PurR [3] ☐ AquaGuard [4] ☐ WaterGuard [5] ☐ Don't know [6]
☐ Other chemical or additive, specify__________________________________ [7]

8. What kind of facility does your household most commonly use to dispose off human fecal waste? [Show pictures to confirm the identity of the facility used.

☐ Flush toilet [1] ☐ Pour flush toilet [2] ☐ Ventilated improved pit (VIP) latrine [3] ☐ Traditional pit toilet [4] ☐ Bush [5]
☐ Field [6] ☐ Ground [7] ☐ Stream [8] ☐ No facility [9] ☐ Other, specify________________________________ [10]

9. When do you usually wash your hands? [Tick all that applies. Do not probe.]

☐ Before eating [1] ☐ After handling domestic animals [2] ☐ Before cooking [3] ☐ After cleaning child who defecated [4]
☐ Before you nurse or prepare baby's food [5] ☐ After you defecate [6] ☐ Never [7] ☐ Other, specify________________ [8]

10. When you wash your hands, what do you usually use? [tick only one]

☐ Water only [1] ☐ Water and soap [2] ☐ Water and ashes [3] ☐ Water and mud or clay [4]
☐ Other, please specify____[5]
