# Supplementary material for: The influence of maternal psychosocial circumstances and physical environment on the risk of severe wasting in rural Gambian infants: a mixed methods approach
Source: BMC Public Health. 2018 Jan 6;18:109. doi: 10.1186/s12889-017-4984-2 (PMC5756408; doi:10.1186/s12889-017-4984-2)
Supplement: Supplementary file 3 — Tool for assessment of maternal mental health (PDF 226 kb) [file 12889_2017_4984_MOESM3_ESM.pdf]

Medical Research Council Unit, The Gambia

Evaluation of the Risk Factors for Malnutrition in Children Recruited to a Supplementation Trial in Rural

Modified Edinburgh Depression Scale

Version 01, 23rd June

MRC | The Gambia Unit

WestKiang No: #Name?

Study ID:

Visit Date: |__|__|/|__|__|/|__|__|__|__|

Completed by: |__|__|

We would like to know something about how you feel in your day-to-day life. Please answer each question

N'lafita alonna nying tiliwoorowulo kono e'kee e'fan jee n'yadiile

Questions and filters	Coding Categories	
1. Have you been able to laugh and see the funny side of things?<br>***Nying tiliwoorowulo kono e'ka meng jee a'kee e'jelendi le ban?***	As much as you always could [0]<br>Not quite so much as you used to [1]<br>Definitely not so much as you used to [2]<br>Not at all [3]	
2. Have you looked forward with enjoyment to things<br>***Nying tiliwoorowulo kono e'ka meng jee e'ka diyaa yea le ban***	As much as you used to [0]<br>A bit less than you used to [1]<br>Definitely less than you used to [2]<br>Hardly at all [3]	
3. Have you blamed yourself unnecessarily when things went wrong?<br>***Nying tiliwoorowulo kono e'ka efang jalailay ban niing yea kukee meng mang nyang***	Yes, most of the time [3]<br>Yes, some of the time [2]<br>Not very often [1]<br>Not at all [0]	
4. Have you been anxious or worried for no good reason<br>***Nying tiliwoorowulo kono e'ka efang jalailay ban niing yea kukee meng mang nyang***	No, not all [0]<br>Hardly ever [1]<br>Yes, sometimes [2]<br>Yes, very often [3]	
5. Have you felt scared or panicked for no good reason?<br>***Nying tiliwoorowulo kono e'ka siila le ban walla foo e'kijoo ke e'faraa asaataree daalila taala.***	Yes, quiet a lot [3]<br>Yes, sometimes [2]<br>No, not much [1]<br>No, not at all [0]	
6. Have you been coping with your daily routine?<br>***Nying tiliwoorowulo kono e'ka sawaree e'lla kowoolo kela le ban.***	Yes, I have been coping as well as ever [0]<br>Yes, most of the times I have coped quite well [1]<br>No, sometimes I have not coped as well as usual [2]<br>No, most of the time I have not been able to cope at all [3]	
7. Have you had difficulty sleeping?<br>***Nying tiliwoorowulo kono seenoo ka koleeya e'maa le ban.***	Yes, most of the time [3]<br>Yes, quiet often [2]<br>Not very often [1]<br>No, not at all [0]	
8. Have you felt sad?<br>***Nying tiliwoorowulo kono yea niikuya soto le ba***	Yes, most of the time [3]<br>Yes, quiet often [2]<br>Not very often [1]<br>No, not at all [0]	
9. Have you been so unhappy that you have been crying?<br>***Nying tiliwoorowulo kono yea niikuya soto le ban, foo e'ka kunboo.***	Yes, most of the time [3]<br>Yes, quiet often [2]<br>Not very often [1]<br>No, never [0]	
10. Do you like to be by yourself?<br>***Nying tiliwoorowulo kono e'ka laafii taree la e'fang maa le ban.***	Yes, most of the time [3]<br>Yes, quiet often [2]<br>Not very often [1]<br>Not at all [0]	

Total Score:
