# Supplementary material for: The influence of maternal psychosocial circumstances and physical environment on the risk of severe wasting in rural Gambian infants: a mixed methods approach
Source: BMC Public Health. 2018 Jan 6;18:109. doi: 10.1186/s12889-017-4984-2 (PMC5756408; doi:10.1186/s12889-017-4984-2)
Supplement: Supplementary file 4 — Tool to support interviews with mothers (DOCX 68 kb) [file 12889_2017_4984_MOESM4_ESM.docx]

**Medical Research Council Unit, The Gambia**

**Evaluation of the risk factors for malnutrition in children recruited to a supplementation trial in rural Gambia**

**In depth interview guide**

Version 06, 11^th^ June 2015

**[Before turning on the recorder]**

- Introduce yourself
- Go through the information leaflet and consent form (consent form completed prior quantitative questionnaires)
- Go over areas to cover
- Explain that you will note down anything that you want to come back to
- Reiterate that there is no right or wrong answer
- That all the information that she gives will be kept confidential and will only be shared with those involved in the study
- That the data collected will be anonymised so they will not be identified
- That any information that she provides that indicates she or someone else is at risk of harm will have to be shared with the relevant health care providers (MRC Keneba doctor/nurse) for hers and the child’s or other adult’s safety.

**Introduction:** I have come back to discuss with you in more details the issues around your experiences of looking after an infant (child under 12 months) in this community.

**Parenting practices**

**Who looked after your baby when they he/she was little – aged 0 to 12 months?**

- Were you the main carer?
- What felt like the biggest responsibilities? First few months, and as they grew older?
- Did anything limit your ability to care for your child during infancy?
- Did any other women in the home you were living in help you with looking after the baby when he/she was very young? Who and in what way? (eg advice, looking after the baby, feeding the baby). Did that change as the baby got older?
- What about any women outside the home? (same probes as above).
- Would you have liked more support? From whom? Why?
- What about the father?

**Is your experience typical of other mothers in the area do you think? What are the differences and why?** (eg not having co-wives? Being single/divorced?)

**Infant feeding practises**

**What did you feed your baby in their first year of life, and how did this change from when they were born up to when they were 12 months old**?

- Did you breastfeed? Why types of other liquids and foods did you introduce when? Why did you add foods? When did breastfeeding stop and why?
- Did you get advice on feeding your baby? Who advised you? What kind of advice and was it useful?
- Did you get any other practical help in feeding your baby? What kind of help, and who from?
- Did health workers give you any advice? Example of useful advice and not so useful advice?
- What factors prevented you from adhering or enabled you to adhere to this advice?

**Breastfeeding under 6 months of age**

- How did you cope with doing this as well as all your other duties?
- How often did you feed you feed your baby? Who determined this frequency, you or the baby? How?
- How did you decide when to stop the feed?
- Where there moments when you were not able to feed your baby? What did you do?

**Would you see what you were doing as the ideal way to feed a baby in this area**?

- What makes you say that*?*
- When should breastfeeding stop and how?
- What types of foods should be introduced when? How often should those foods be consumed?

**Is your experience typical of other mothers in the area do you think? If not what are the differences and why?**

- Does amount of money or types of sources of income in the household play a role? How?
- Do family relationships and amount of support play a role? How?

**Handling death**

- Have you or anyone close to you experienced the death of a child?
- How did you feel?
- How did you/they cope? Has it affected how you/they look after infants? In what way?
- What support did you get? Was it helpful?

*Death of the husband*

- Have you or anyone close to you experienced the death of your/a husband?
- How did you feel?
- How did you/they cope? Has it affected how you/they look after infants? In what way?
- What support did you get? Was it helpful?

**Perinatal stressors and education**

- What areas of your life caused you to worry or become distressed during pregnancy with child ……?
- How about when you delivered child …..?
- How about during the first year of child …..’s life?
- Did it make it difficult for you to care for child …..? How?
- Did this affect your ability to go about your day to day activities? How?
- How did you cope with these worries? Did anyone help you? Who?
- Do you think that educating a mother (Arabic school/English school) has any effect on her ability to care for her infant? How?
- How about for you, do you think education/lack of it has affected you and your family? How?

**The household environment/hygiene**

**Does the general cleanliness in the home environment affect the health and growth of an infant? In what way?**

- What about where food is kept and stored, and how it is prepared and served?
- Availability of clean water and good toilet facilities?
- Disposal of the faeces of infants and children?
- Hand washing by the carer? Hand washing of the infant?

**What limits mothers in this area from achieving some of these things? Which aspects are hardest to achieve? Which ones are most important to achieve?**

- Any examples from your household of strengths or challenges?

Is there anything you would like to ask me?

Thank you for your time

**[Turn off the recorder]**

**Debrief**

- Inform the participant that findings will be fed back to the community and made public after the study has been completed

Once the participant has left or you have left the participant’s compound, please make **field notes**:

- Any notable themes
- Social characteristics of setting
- Participant characteristics
- Your perception of the person/thoughts/emotions
- Notable events during interview
- Note any suggested changes to the topic guide
