# Supplementary material for: The influence of maternal psychosocial circumstances and physical environment on the risk of severe wasting in rural Gambian infants: a mixed methods approach
Source: BMC Public Health. 2018 Jan 6;18:109. doi: 10.1186/s12889-017-4984-2 (PMC5756408; doi:10.1186/s12889-017-4984-2)
Supplement: Supplementary file 5 — Tool for to support interviews with fathers (DOCX 67 kb) [file 12889_2017_4984_MOESM5_ESM.docx]

**Medical Research Council Unit, The Gambia**

**Evaluation of the risk factors for malnutrition in children recruited to a supplementation trial in rural Gambia**

**In depth interview guide**

Version 02, 11^th^ June 2015

**[Before turning on the recorder]**

- Introduce yourself
- Go through the information leaflet and consent form (consent form completed prior quantitative questionnaires)
- Go over areas to cover
- Explain that you will note down anything that you want to come back to
- Reiterate that there is no right or wrong answer
- That all the information that she gives will be kept confidential and will only be shared with those involved in the study
- That the data collected will be anonymised so they will not be identified
- That any information that she provides that indicates she or someone else is at risk of harm will have to be shared with the relevant health care providers (MRC Keneba doctor/nurse) for hers and the child’s or other adult’s safety.

**Introduction:** I have come back to discuss with you the issues around your experiences of looking after an infant (child under 12 months) in this community.

**Parenting practices**

**Who looked after your baby when they he/she was little – aged 0 to 12 months?**

- Who was the main carer?
- Were you able to help in the care of this child during these early years?
- What help did you give? In your view was it enough?
- Who else helped?
- What felt like the biggest responsibilities? First few months, and as they grew older?
- What did you feel your role was as the father?

**Is your experience typical of other fathers in the area do you think? What are the differences and why?** (eg having more than one wife? Being divorced?)

**Infant feeding practises**

**How was your baby fed in their first year of life, and how did this change from when they were born up to when they were 12 months old**?

- Did you know how your baby was fed? Did his/her mother breastfeed them? Were any other feeds or fluids introduced? When?
- Were you involved in the decision made around how they were fed?
- What did you feel your role was in these decisions?
- In your view did your child receive the right type of feeds for their age? What makes you say this?
- What factors do you think ensured that your child received the right feeds/prevented your child from receiving the right feeds?

**Is your experience typical of other fathers in the area do you think? If not what are the differences and why?**

- Does amount of money or types of sources of income in the household play a role? How?
- Do family relationships and amount of support play a role? How?

**Handling death**

- Have you or anyone close to you experienced the death of a child?
- How did you/they cope?
- How did your wife cope?
- What support did you get as a family? Was it helpful?
- How has this affected your involvement in the care of your children? In what way?

*Death of the husband*

- Does the death of a husband affect the growth of young children (under 1 year)? How?
- How do women cope with this?
- How does the family or community support women whose husbands have passed away?
- Is this helpful?

**Stressors and education**

- What areas of your life caused you to worry or become distressed during the pregnancy of child ....?
- How about soon after child ....was born?
- How about in the first year child...’s life? How did you cope?
- Do you know what worried or distressed your wife during pregnancy or after the birth of child ...? Did she cope? How?
- How about during the first year of child …..’s life?
- How did this affect your involvement in the care of your child?
- How about your wife, did it make it difficult for her to care for your child …..? How?
- How did she cope with these worries? Did anyone help her? Who?
- Do you think that educating a mother (Arabic school/English school) has any effect on her ability to care for her infant? How?
- How about for you, do you think education/lack of it has affected you and your family? How?

**The household environment/hygiene**

**Does the general cleanliness in the home environment affect the health and growth of an infant? In what way?**

- What about where food is kept and stored, and how it is prepared and served?
- Availability of clean water and good toilet facilities?
- Disposal of the faeces of infants and children?
- Hand washing by the carer? Hand washing of the infant?

**What limits carers in this area from achieving some of these things? Which aspects are hardest to achieve? Which ones are most important to achieve?**

- Any examples from your household of strengths or challenges?

Is there anything you would like to ask me?

Thank you for your time

**[Turn off the recorder]**

**Debrief**

- Inform the participant that findings will be fed back to the community and made public after the study has been completed

Once the participant has left or you have left the participant’s compound, please make **field notes**:

- Any notable themes
- Social characteristics of setting
- Participant characteristics
- Your perception of the person/thoughts/emotions
- Notable events during interview
- Note any suggested changes to the topic guide
